# Context aware semantic adaptation network for cross domain implicit sentiment classification

**DOI:** 10.1038/s41598-021-01385-1

**Published:** 2021-11-11

**Authors:** Enguang Zuo, Alimjan Aysa, Mahpirat Muhammat, Yuxia Zhao, Kurban Ubul

**Affiliations:** 1 College of Information Science and Engineering, Xinjiang University, Urumqi, 830046 China; 2 Xinjiang Multilingual Information Technology Key Laboratory, Urumqi, 830046 China; 3 School of Mathematics and Computer Applications Shangluo University, Shangluo, 726000 China

**Keywords:** Computational science, Computer science, Information technology, Machine learning

## Abstract

Cross-domain sentiment classification could be attributed to two steps. The first step is used to extract the text representation, and the other is to reduce domain discrepancy. Existing methods mostly focus on learning the domain-invariant information, rarely consider using the domain-specific semantic information, which could help cross-domain sentiment classification; traditional adversarial-based models merely focus on aligning the global distribution ignore maximizing the class-specific decision boundaries. To solve these problems, we propose a context-aware semantic adaptation (CASA) network for cross-domain implicit sentiment classification (ISC). CASA can provide more semantic relationships and an accurate understanding of the emotion-changing process for ISC tasks lacking explicit emotion words. (1) To obtain inter- and intrasentence semantic associations, our model builds a context-aware heterogeneous graph (CAHG), which can aggregate the intrasentence dependency information and the intersentence node interaction information, followed by an attention mechanism that remains high-level domain-specific features. (2) Moreover, we conduct a new multigrain discriminator (MGD) to effectively reduce the interdomain distribution discrepancy and improve intradomain class discrimination. Experimental results demonstrate the effectiveness of different modules compared with existing models on the Chinese implicit emotion dataset and four public explicit datasets.

## Introduction

Sentiment analysis is considered one of the fundamental problems in natural language processing (NLP), and with social media developing rapidly, it is widely applied in real scenarios such as comment analysis, food safety monitoring, and public opinion mining. Such tasks are usually defined as identifying the emotional polarity (e.g., positive, negative, or neutral) of a given text, sentence, or aspect.

The expression of emotion can be explicit or implicit. The implicit expression of emotions is defined as ’A language fragment (sentence, clause or phrase) that expresses subjective sentiment but contains no explicit sentiment word’^1,2^. We exploit the following examples to show the difference of two expressions:

*Explicit:*你做的蛋炒饭太好吃了,我很喜欢! *(English translation: “The rice fried with eggs is so delicious; I* like *it very much!” label=positive)*

*Implicit:*这家蛋炒饭有种妈妈的味道! *(English translation: “The rice fried with eggs in this restaurant reminds me of my mother!” label=positive)*

Example 1 uses the word ‘喜欢 (like) ’ to show a clear positive tendency. To express their views, people also use Example 2 (e.g., metaphor, sarcasm). In this sentence, no explicit emotional words are used and the individual’s emotional tendency is embedded in the semantic meaning of the text. This phenomenon creates an exceptional challenge for implicit sentiment classification tasks. Moreover, accompanied by the absence of a large-scale labeled corpus, even with an advanced deep learning model, the classification accuracy of implicit sentiment classification tasks is not ideal^3^.

**Figure 1:**
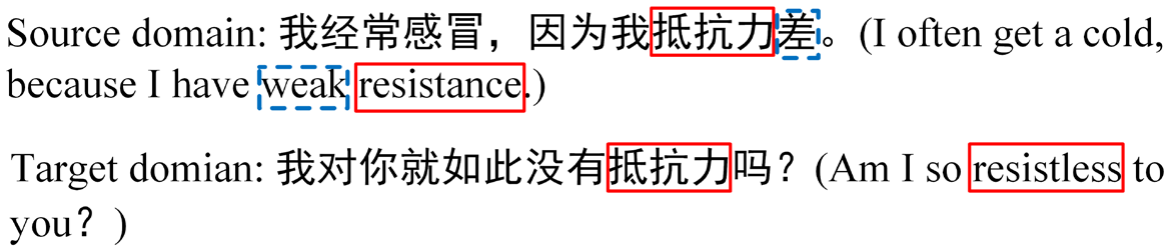
Example of domain-invariant and domain-specific. The sentiment expressions marked by red lines are virus domain-specific, while the broken blue lines marked domain-invariant.

**Figure 2:**
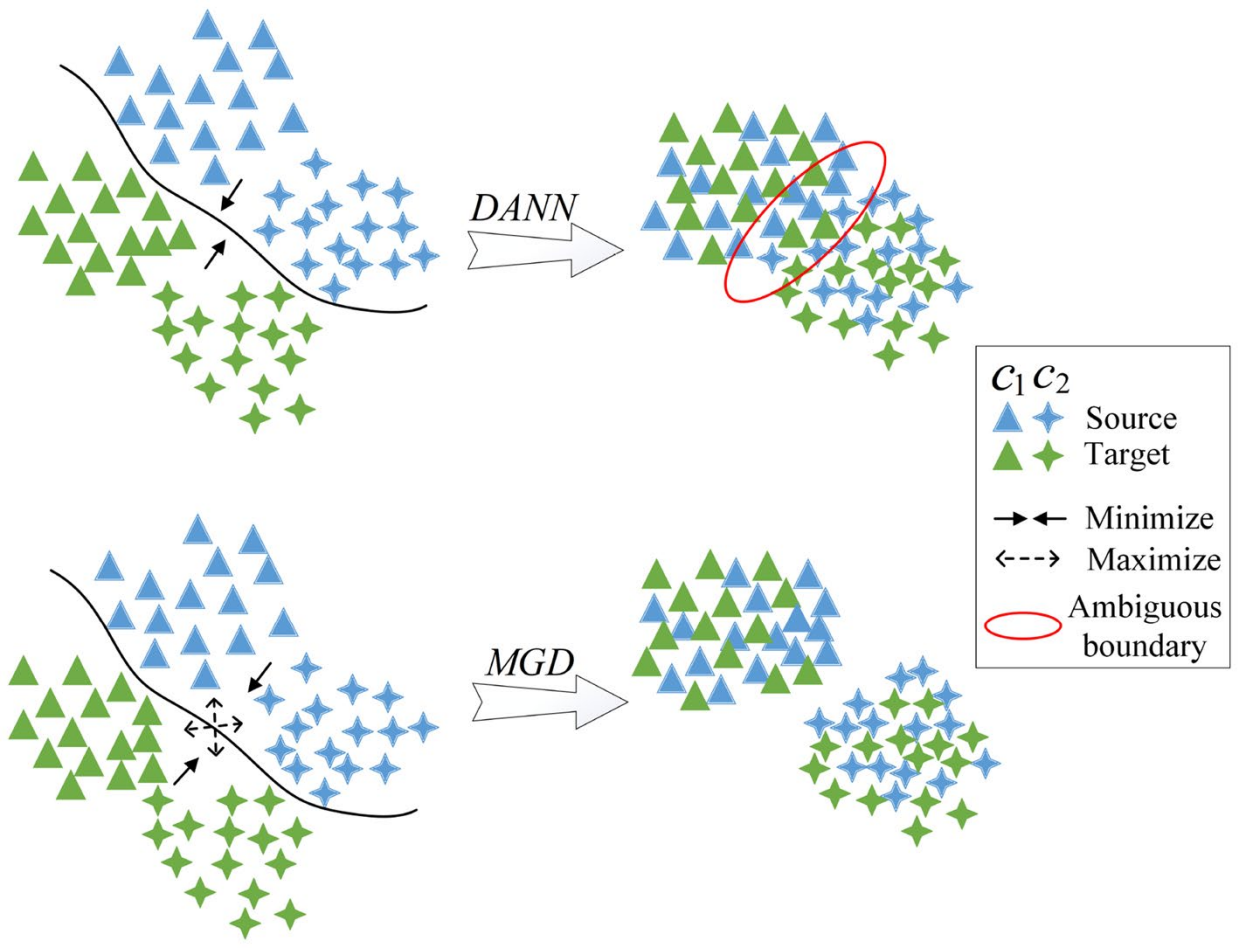
A comparison between the traditional domain discriminator DANN and the proposed MGD, where minimization means that the distribution difference is minimized in different domains and maximization means that the distribution difference is maximized between different classes, which come from different domains.

One solution to this problem is cross-domain sentiment classification, which aims to exploit the rich labeled data in the source domain, e.g., explicit sentiment corpus, to help the sentiment analysis task in another domain lacking for or even without labeled data, e.g., implicit sentiment corpus. Recently, relevant models on cross-domain sentiment classification have mainly focused on learning domain-invariant features whose distribution is similar in the source and target domains^4^. These methods attempt to reduce the discrepancy between domain-specific latent feature representations. Inspired by this idea, most existing adversarial-based methods, e.g., domain adversarial neural network (DANN) ]^5^, reduce feature differences by fooling a domain discriminator and have achieve promising results^6,7^. However, to achieve explicit-to-implicit positive transfer, these methods still have two major inherent drawbacks that need to be addressed:

- Existing studies mostly focus on learning the domain-invariant information (e.g. ‘喜欢 (like)’, ‘坏 (bad)’, ‘差 (weak)’), rarely consider the usage of domain-specific semantic information (e.g. ‘蛋炒饭 (the rice fried with eggs)’, ‘抵抗力 (resistance)’), which is also helpful for cross-domain implicit sentiment classification^7,8^. Figure 1 shows that when the source domain-specific words appear in the target domain, the semantic knowledge learned from the source domain helps the target domain classification.
- Traditional adversarial-based models merely minimize the marginal distribution of the two domains and ignore maximizing the class-specific decision boundaries. As shown in Fig. 2*(DANN)*, the features near the decision boundary may be ambiguous and even tangled together with traditional domain discriminator training, thus blocking adaptation performance.

To tackle the above limitations identified above, we aim to use graph convolutional networks (GCNs). GCNs have a multilayer architecture, with each layer aggregating the information of nodes in the graph structure using features of immediate neighbors. Nevertheless, sequential free texts are unstructured data. Therefore, GCN-based text learning must conduct graph representation learning from the free text before graph convolution. Different from sequence learning models, GCNs can directly represent complex structured data. A GCN has the potential to capture domain-specific semantical information with GCN layers. Recently, GCN models have gained widespread attention and have been successfully deployed on text-word relationships^9–11^, and explicit sentiment analysis^12,13^. However, these graph-based models only considered intrasentence hierarchical dependency relationships and ignored intersentence semantic associations.

Therefore, in this paper, we propose a novel context-aware semantic adaptation network (CASA) for cross-domain implicit sentiment classification via GCNs. To obtain inter- and intrasentence semantic associations, we build a context-aware heterogeneous graph (CAHG). CAHGs build graphs in each document by regarding tokens and sentences as nodes (hence heterogeneous graph). The intrasentence propagation is constrained by the syntactic dependency tree, and intersentence propagation is constrained by the sentence-free sequence and term frequency-inverse document frequency (TF-IDF) local token cooccurrence information. The information propagates inter- and intrasentences via the GCN layers, followed by an attention mechanism that keeps high-level domain-specific features. We also conduct a multigrain discriminator (MGD) which is imposed during domain adaptation to minimize domain distribution and maximize class identification. The domain adaptation layer makes the source domain and target domain inseparable e through adversarial training, reduces the representation distribution gap between source and target domain data in a coarse-grained manner. The class adaptation layer utilize a classifier to judge different domain samples, whether class consistent, to distinguish samples in different classes. Figure 2 illustrates the difference between the traditional domain adversarial method of domain-adversarial training of DANN^5^ and MGD.

In short, the main contributions of this paper are as follows:

1. We proposed a new transfer learning model CASA for the implicit sentiment classification task via GCNs, which is the first attempt to transfer explicit sentiment information to implicit sentiment.
2. Our model provides a context heterogeneous graph, which can effectively extract inter- and intrasentence semantic information. Moreover, CASA improved the model’s generalization ability for implicit classification tasks and increased each sentiment’s polarity discrimination in the domain.
3. We evaluate CASA on the Chinese implicit sentiment analysis dataset (SMP-ECISA 2019). CASA outperforms existing models in four different source domains. We also provide a visualization to demonstrate that CAHG can capture domain-specific information, and MGD can make features near decision boundaries more distinguishable.

## Related works

### Sentiment analysis

The existing sentiment calculation and sentiment analysis methods can be divided into three categories: knowledge-based methods, statistical methods and hybrid methods^14^.

Knowledge-based methods are popular because of their simplicity and ease of use, but their effectiveness is limited mainly by the depth and breadth of the established knowledge base. Statistical methods based on machine learning and deep learning have been widely used in Chinese sentiment classification, such as fine-grained sentiment analysis framework^15^, multi-label sentiment analysis model^16^, aspect-level sentiment analysis research based on Reinforcement Learning^17^, etc. On the other hand, more and more scholars have realized the particularity of Chinese characters and tried to model Chinese radicals^18–22^. The hybrid method aims to describe the rules of emotional expression better and realize the machine’s perception of semantics^23^. The model CASA in this paper considers contextual semantic perception and introduces cross-domain explicit emotional knowledge.

Attention mechanism shows good performance in sentiment analysis tasks. It improves the interpretability of neural networks by letting people know the location of the focus^24–27^. They exemplified recent research on attention-based sentiment analysis.

Various sentiment analysis tasks usually focus on realizing binary classification (positive and negative classification), which cannot better describe emotions. In contrast, Wang proposed a multi-level emotion perception method with contradiction processing^28^. However, these sentence-level sentiment analyses cannot be directly applied to implicit sentiment tasks because, in implicit sentiment tasks, the emotion of the target sentence holds different polarities for different contexts.

### Implicit sentiment analysis and GCNs

Models based on RNNs^29,30^ and CNNs^31^ in deep neural networks are widely used in sentiment classification tasks. In RNN-based models, an attention mechanism is usually introduced because each word in the text contributes differently to the classification task^32–34^. CNN-based models^35,36^ use character-level CNNs to extract semantic information from text. However, these models lack an effective mechanism to capture the information in the dependency tree structure. In works of^37–39^, structural and semantic information extracted from the tree structure of sentences, such as a dependency tree or grammar tree by LSTM or BiLSTM, was used for the sentiment classification task. Although Tree-LSTM can extract more accurate semantic information from text, it is difficult to perform parallel computing and requires a longer time to train. After that, CNNs were introduced into the tree structure information encoding process by^40,41^. In their work, a phrase structure tree and syntax-dependent tree were used to encode the semantic information of the target sentence and the context, respectively. However, in the above tree-based convolutional neural network model, sentences are considered to be independent of each other, so information regarding the relationship between sentences is lost. To solve this problem, our model extracts semantic information via GCNs^9^.

GCN models have attracted widespread attention and have been successfully deployed in NLP tasks. Yao et al.^10^ builds a corpus-level text graph by word-word co-occurrence and document-word relations for text classification. Zhang et al.^42^ introduces a TreeGCN, where the GCN is used to encode the dependency syntactic structure. Zhang et al.^12^ presented an aspect-based GCN to demonstrate that GCNs can achieve long-range word dependency. Zhang et al.^43^ employ gated graph neural networks document-level graph word interaction. In contrast to their works, we regard the tokens and sentences in each document as graph nodes. The graph maintains inter- and intrasentence constraints to capture semantic information. It can obtain more accurate text semantics while increasing the interpretability of the model.

### Transfer learning in sentiment analysis

Even with a strong deep learning model, the classification accuracy of implicit emotion classification problems is not ideal in the absence of sufficient labeled data^34,41^. Yosinski et al.^44^ discusses the application of transfer learning in deep neural networks for single domains. It draws an important conclusion: adding fine-tuning will improve the performance of the deep transfer network. In the cross-domain scenario, the difference between the probability distribution of the source domain’s data and the target domain’s data is significant. The use of fine-tuning alone may lead to negative transfer. The purpose of domain adaptation, also known as cross-domain learning, is to reduce the difference in distribution representation of the data of the source and target domains. In domain adaptation, a direct way to solve the above problem is to use a certain distribution distance measurement method to measure the distance between distributions and reduce the distance in the model training process. However, the calculation of distance measurements is difficult. Many methods based on domain adversarial models have been proposed^5,6^.

However, existing domain adversarial models for sentiment analysis focus on explicit sentiment classification or aspect-level sentiment classification^45,46^ without considering implicit situations. Due to data scarcity and the task’s value, transfer learning is more urgent for implicit sentiment analysis. To the best of our knowledge, CASA is the first explicit-to-implicit transfer learning model. MGD improved the model’s generalization ability to implicit emotion classification and the discrimination of each sentiment polarity.

**Figure 3:**
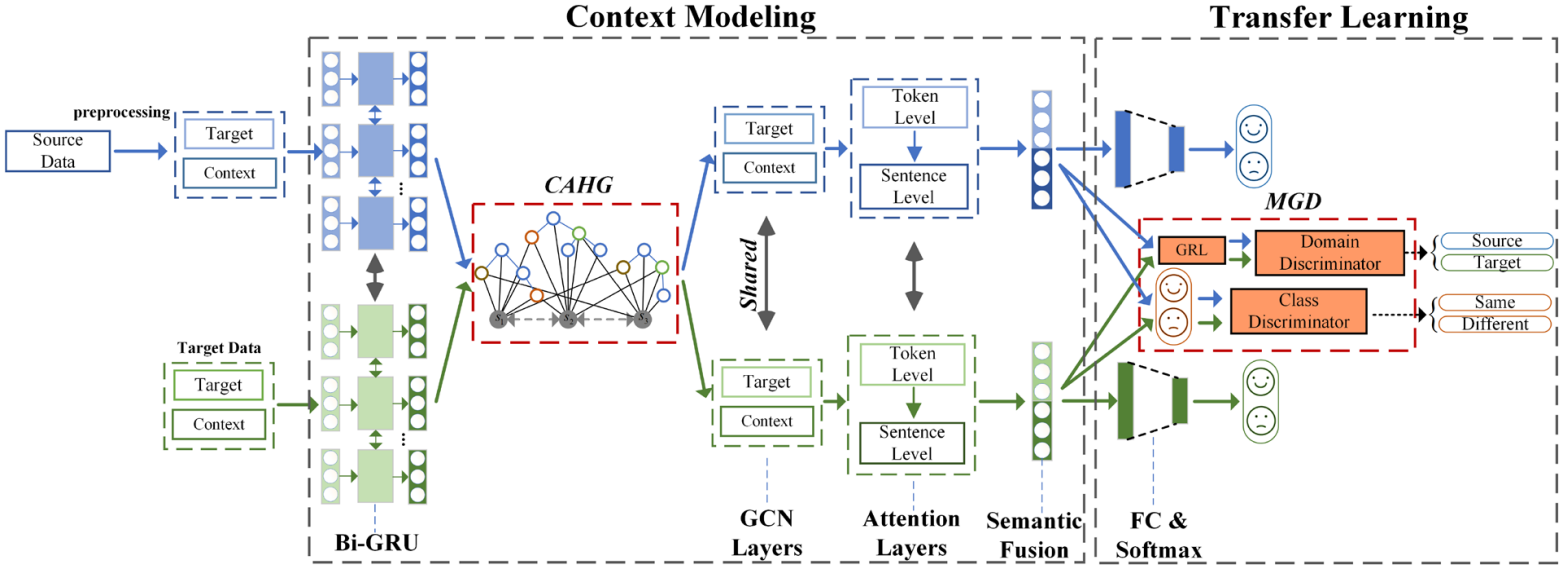
Overview of the context-aware semantic adaptation network. CAHG represents the context background structure in both the source and target domains. Attention layers are hierarchical attention, token-level of target sentence, and sentence-level of context background. There are two layers of GCNs. MGD represents the proposed multigrain discriminator. Details on the CAHG and MGD are discussed in later sections.

## Methods

### Problem definition and approach overview

#### Domain definition

A domain *D* consists of marginal distribution *P*(*x*) and m-dimensional feature space *X*, namely $D= \lbrace P(x), X\rbrace$.

#### Task definition

Given the domain *D*, the task is composed of a classifier *f*(*x*) and a class label set *Y*, namely $T=\lbrace f(x), Y \rbrace$, where $f(x)=Q(y|x)$ represent the conditional probability distribution, $y \in Y$.

#### Purpose

Given a training data come from source domain $D_s = \lbrace (x_i,y_i)\rbrace ^{n_s}_{i=1}$ and target domain $D_t = \lbrace (x_{n_s+i},y_{n_s+i})\rbrace ^{n_t}_{i=1}$, we assume that there is a difference between the probability distribution $P(X^s)$ and $P(X^t)$. under this settings, the purpose is to learn a prediction function $f(x):X \rightarrow Y$ that classify the target examples correctly during testing step.

The overview of the CASA framework is described in Fig. 3. As shown in Fig. 3, the proposed approach is divided into two steps: context modeling and transfer learning. Specifically, we first built CAHG in the context modeling phase to extract the semantic relationships unrelated to explicit and implicit sentiment domains. In the transfer learning step, MGD realizes fine-grained adaptation through a domain discriminator and label consistency discriminator so that the model has stronger generalization ability. We present the details of different components as well as the training process in the following section.

### Bi-GRU encoder

First, we use the bidirectional gated recurrent unit (Bi-GRU)^47^ to encode the text input from the source or target domain to obtain contextualized word-level representation ${\mathbf {H}} = [{\mathbf {h}}_1, {\mathbf {h}}_2,\ldots , {\mathbf {h}}_i,\ldots , {\mathbf {h}}_n]$, where *m* denotes the vector dimension and ${\mathbf {h}}_i \in R^m$ is the hidden layer state vector at time *i*. The reason for employing this layer is to correct the information of the syntactic dependency tree, which was built by HANLP. We use HANLP for this: https://www.hanlp.com.

### GCNs over the context-aware heterogeneous graph

To express the text’s more valuable information, we construct a document-level heterogeneous graph, which retains intrasentence dependency information, and has intersentence relationship representation. We define $G = (V, E)$ as a text graph, where $|V|=n$ is the number of nodes and *V*, *E* represents the node set and the edge set of graph *G*, respectively. We construct *G* based on the token-token dependency relationship, TF-IDF, and sentence order. The token’s TF-IDF determines the edge weight between the sentence node and the token node in the sentence. We define TF as the number of words that appear in the sentence, and IDF as the logarithm of the overall sentence number in the text to the sentence number, which contains the token. The formal definition is as follows formula (1–3).

1 $$\begin{aligned} TF-IDF= & {} TF \times IDF \end{aligned}$$

2 $$\begin{aligned} TF_{w}= & {} \frac{S_{w}}{S} \end{aligned}$$

3 $$\begin{aligned} IDF_w= & {} log\left( \frac{Doc}{Doc_{w}+1}\right) \end{aligned}$$

Among them, $S_w$ and *S* represent the number of token occurrences in the input sentence and the sentence token’s total number, respectively. $Doc_w$ and *Doc* represent the number of sentences in the input text with the token and the total number of sentences, respectively.

**Figure 4:**
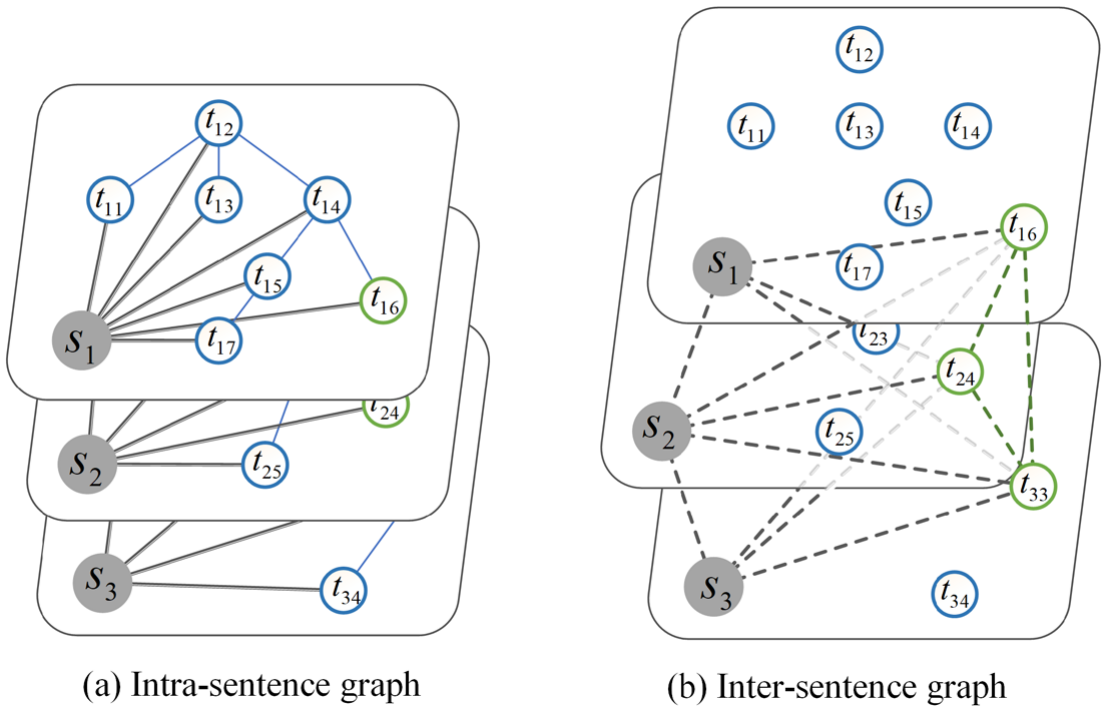
A tony demo of the CAHG. $\hbox {S}_i$ represents for the i-th sentence, $t_{ij}$ represents for the j-th token in the i-th sentence, the intrasentence edges between the tokens are dependency relations, and the green color represents these tokens at least part of the Chinese characters are the same.

Meanwhile, we introduce the sentence sequential features to represent the sentence-sentence relationship. The matrix ${\mathbf {H}} \in R^{m \times n}$ consists of the feature vector ${\mathbf {h}}_i \in R^m$ of *n* nodes, and the adjacency matrix $A \in R^{m \times n}$ is used to represent the weights between nodes in graph *G*. The relationships of the edges between nodes *p* and *q* are formally defined as function (4–5):

4 $$\begin{aligned} A_{p,q}= & {} \left\{ \begin{array}{ll} D(p,q)&{}\text {p,q all tokens} \\ TF-IDF_{p,q}&{}\text {p is sentence, q is token} \\ Order(p,p+1)&{}\text {p is sentence} \\ 0 &{}\text {otherwise} \\ \end{array} \right. \end{aligned}$$

5 $$\begin{aligned} D_{p,q}= & {} \left\{ \begin{array}{ll} Tree(p,q) &{}\text {p,q in same sentence} \\ \frac{N_{syntactic}}{N_{total}}&{} \text { p,q in different sentence} \\ \end{array} \right. \end{aligned}$$

$Order(p,p+1)$ denotes sentence $p \rightarrow p + 1$’s natural reading order in the text. *Tree*(*p*, *q*) is the relationship between token nodes *p* and *q* in the dependency tree. $N_{syntactic}$ represents the number of times that the tokens p and q have a relationship in the current text (the relationship means that p and q in at least part of the Chinese characters are the same), and $N_{total}$ is the number of times that p and q appear throughout the dataset. To facilitate the description of the process of information transfer in the graph, we divide CAHG into two subgraphs in Fig. 4. One of the subgraphs describes the transfer of information within sentences, and the other describes the transfer of information across sentences.

The information propagates inter- and intrasentences via the GCN layers, followed by a hierarchical attention layer that keeps high-level domain-specific features. The final text representation is defined as follows:

6 $$\begin{aligned} \alpha _{t,i}= & {} \frac{\exp \left( \omega _{t}^{\top } \tanh \left( {\mathbf {W}}_{t} {\mathbf {H}}^G_{t,i}+{\mathbf {b}}_{t}\right) \right) }{\sum _{i^{\prime }} \exp \left( \omega _{t}^{\top } \tanh \left( {\mathbf {W}}_{t}, {\mathbf {H}}^G_{t,i^{\prime }}+{\mathbf {b}}_{t}\right) \right) }\end{aligned}$$

7 $$\begin{aligned} {\mathbf {O}}_{t}= & {} \sum _{i} \alpha _{t, i} {\mathbf {H}}^G_{t,i}\end{aligned}$$

8 $$\begin{aligned} {\mathbf {r}}= & {} (\mathbf {O_{t}} \oplus \mathbf {O_{c}}) \end{aligned}$$

In formula (6–8), ${\mathbf {r}}$ is the final representation of the text, which is composed of the target sentence ${\mathbf {O}}_t$ and the relevant context ${\mathbf {O}}_c$. $\oplus$ represents the splice operation. ${\mathbf {H}}_{t}^{G}$ represents the output from two-layer GCNs^5^. Then ${\mathbf {H}}_{t}^{G}$ through a one-layer multilayer perceptron. Finally, ${\mathbf {O}}_t$ is obtained by weighted summation of the MLP output. The sentence-level attention mechanism ${\mathbf {O}}_c$ is a mirror of the word-level attention mechanism.

### Multi-grain discriminator

Ben-David and Ganin proved that to perform domain adaptation to reduce the target domain’s prediction error, maximizing the discriminator error between the source and target is necessary^5,48,49^. Therefore, adversarial-based transfer learning is widely used to solve the domain adaptation problem. Although it has considerable advantages, we observed that the domain discriminator could only reduce the marginal distribution distance.

However, the relationship between marginal distributions and conditional distributions is uncertain. As indicated in^50^, minimizing the difference between conditional distributions is critical to the robustness of distribution adaptation. We propose MGD, which consists of a domain discriminator *T* and an emotional polarity discriminator *D*. *T* makes the source domain and target domain not separable through domain confrontation. *D* maximizes the difference between different labels through label consistency identification. When the class discriminator *D* can accurately identify whether the sample labels from the source domain and the target domain are the same, the model can learn the class invariant features from the two domains. The formula is derived as follows:

9 $$\begin{aligned} G_{T}= & {} softmax({\mathbf {W}}_{T}r_{\cdot }+{\mathbf {b}}_{T}),\mathbf {\cdot } \in (s,t) \end{aligned}$$

10 $$\begin{aligned} L_{T_{i}}= & {} -Y^{D}_{i}log(G_{T}({\mathbf {r}}_{i})-(1-Y^{D}_{i})log(G_{T}({\mathbf {r}}_{i}))) \end{aligned}$$

where $G_{T}$ denotes the domain discriminator, $G_{T}(\cdot )$ is the output prediction labels, $L_{T_{i}}$ is the classification error, $Y^{D}=[s,t]$ is a set of domain labels, and *s* and *t* represent the source and target domains, respectively.

To maximize the extraction of domain invariant features, we hope to maximize the discrimination error $G_{t}$. As suggested in^5^, this min-max game is implemented by a gradient reversal layer. Specifically, when the network is undergoing the gradient back-propagation process, we will change $\nabla L_{T}$ into $-\eta \nabla L_{T}$. $\eta > 0$ is a controllable hyper-parameter.

11 $$\begin{aligned} G_{D}= & {} softmax({\mathbf {W}}_{D}\mathbf {(r}_{s} \oplus {\mathbf {r}}_{t})+ {\mathbf {b}}_{D}) \end{aligned}$$

12 $$\begin{aligned} L_{D_{i}}= & {} -I log(G_{D}({\mathbf {r}}_{i}))-(1-I log(G_{D}({\mathbf {r}}_{i})) \end{aligned}$$

where *I* denote an indicator function:

13 $$\begin{aligned} I = \left\{ \begin{array}{ll} 1 &{} label({\mathbf {r}}_s)=label({\mathbf {r}}_t) \\ 0 &{} label({\mathbf {r}}_s)\ne label({\mathbf {r}}_t) \\ \end{array} \right. \end{aligned}$$

Here, $G_{D}$ denotes the class-consistency discriminator, $G_{D}(\cdot )$ is the output prediction labels, and $L_{D_{i}}$ is the classification error. Note that as $L_{D}$ drops, the distribution difference also decreases in the same sentiment polarity between different domains. Finally, $G_{D}$ enhances the identifiability of each sentiment polarity.

### Sentiment classification and training

The vector ${\mathbf {r}}$ obtained by the feature extractor is sent to the cascade of the fully connected layer and softmax layer to generate class distributions. The formula is as follows:

14 $$\begin{aligned} P= softmax({\mathbf {W}}_{p}{\mathbf {r}}+ {\mathbf {b}}_{p}) \end{aligned}$$

where $P \in R^C$ represents the predicted soft distribution, *C* is the number of classifications, and $\mathbf {W_p} \in R^{c \times m}$ and $b_{p}$ represent training weights and offsets. The cross-entropy has a loss function, given as:

15 $$\begin{aligned} L_C=\sum _{i \in D}\sum _{j=1}^{C}Y_{ij}logP_{ij} \end{aligned}$$

*D* is the document’s index with the label, and *Y* is the real label matrix. Therefore, we can obtain the classification loss of the source domain $L_{C_{s}}$ and the classification loss of the target domain $L_{C_{t}}$.

16 $$\begin{aligned} D_{KL}(P_s\parallel P_t)=\sum _{i=1}^{N^s}\sum _{c}^{C}p_{s}^{c} ({\mathbf {r}}_i)log\left( \frac{p_{s}^{c}({\mathbf {r}}_i)}{p_{t}^{c}({\mathbf {r}}_i)}\right) \end{aligned}$$

Before the training stage, we were motivated by^42^, who proposed mutual learning in supervised single-domain tasks. The Kullback Leibler divergence of the predicted source class distribution and predicted target class distribution is calculated and vice versa. The two KL divergences measure the similarity of the two distributions. Finally, the overall loss function in CASA consists of both source and target loss, which are given as follows: $L_{s}=L_{C_{s}}-\eta _{T}L_{T_{s}} + \eta _{D}L_{D_{s}}+\lambda D_{KL}(P_s\parallel P_t)$ and $L_{t}=L_{C_{t}}-\eta _{T}L_{T_{t}} + \eta _{D}L_{D_{t}}+\lambda D_{KL}(P_t\parallel P_s)$. Here, $\eta _{T}$, $\eta _{T}$ and $\lambda$ are hyperparameters.

## Experiments

### Datasets and evaluation indicator

Our target domain data set is The Evaluation of Chinese Implicit Sentiment Analysis task in SMP2019 (one of the top academic conferences on social media processing in China). The dataset contains two types of content in each document: context and target sentence. We chose four benchmark datasets of explicit sentiments as the source domain. They are the Weibo-60000 dataset, the hotel review dataset, the SMP-2020’s virus Weibo dataset, and the SMP-2020’s general Weibo dataset. The data is available at: http://biendata.com/competition/smpecisa2019/, http://www.pudn.com/Download/item/id/3993718.html, http://www.searchforum.org.cn/tansongbo/corpus1.php, https://smp2020.aconf.cn/.

To clean the dataset, we performed some preprocessing. The construction of a heterogeneous graph requires contextual sentence nodes and a dependency tree structure. We performed the following preprocessing, and Table 1 summarizes the statistics:

1. To keep intact the dependent syntax structure, filter out sentences, which have no subject-predicate structure.
2. As suggested by^41,51^, sentiment polarity consistency exists between the context semantic background and the target sentence. To match the target domain data granularity, we randomly select a sentence in each source domain document as the target sentence and the rest as the context.

**Table 1:** Statistics of the target domain and source domain datasets.

Dataset		Documents	AvgLength	T2TEdges	S2SEdges	S2TEdges	Classes
**Explicit**	Virus	7146	35.46	63.47	2.11	44.67	2
	Usual	22019	39.37	64.8	1.85	45.62	2
	Weibo	119999	40.99	72.32	2.83	51.34	2
	Hotel	9998	88.78	187.03	5.02	129.7	2
**Implicit**	SMP_2019	9733	51.81	111.51	4.44	78.95	2

The AvgLength denotes the average number of tokens in each text; the T2TEdges, the S2SEdges, and the S2TEdges indicate the average number of times token-token, sentence-sentence, sentence-token edges occurrences in each text, respectively.

We compute every model classification accuracy and F1 score in test dataset as evaluation indicator. The F1 score calculation and accuracy are shown as follows:

17 $$\begin{aligned} F1_{i}= & {} \frac{2 \times P_i \times R_i }{P_i +R_i } \end{aligned}$$

18 $$\begin{aligned} \text {Accuracy }= & {} \frac{\left| P(x)=Y(x) \subseteq x: x \in D_{t}\right| }{\left| x: x \in D_{t}\right| } \end{aligned}$$

where $P_i$ and $R_i$ mean the precision and recall of i-th sentiment polarity. After the above equation, we can through calculate metrics for each label, and find their unweighted mean getting macro-F1 score, i.e. $\text {macro-F1}=\frac{1}{N}\sum _{i \in N_i}F1_{i}$. *P*(*x*) is the predicted label and *Y*(*x*) is the actual label of sample *x*, respectively.

### Models for comparison

To fully verify and understand CASA, we divide the baseline models into two groups for comparison:

#### Non-transfer

To demonstrate the benefits from heterogeneous graphs, we compare with the following methods without transfer:

- *TextCNN*^31^, *TextRNN*^52^ and *BiLSTM*$+$*Att*^53^: These are the basic deep neural networks in sentiment classification.
- *TreeLSTM*^54^: An LSTM network based on a tree structure, which solves the problem of the emotional classification of nonlinear systems such as dependent trees.
- *TreeGCN*^39^: BiLSTM is used to encode the input word vector to obtain the hidden state with context information. It then uses a GCN convolution to obtain the neighboring node information, which enhances the GCN’s robustness.
- *CASA-T-D*: The CASA feature extractor part for examining the ability of CAHG to express text information.

#### Transfer

To investigate the effectiveness of each part in the CASA, we also compare the following frameworks for experiments. For a fair comparison, we use CASA-T-D as a feature extractor in other methods.

- *Fine-tuning*: Initialize the CASA-T-D parameters randomly, then train on the source domain dataset, and finally fix the parameters and fine-tune the model in the target domain dataset.
- *DANN*$$^+$$^5^: The model adopts the idea of domain adversarial, which has a feature extractor and a domain discriminator.
- *CCSA*^55^: A unified framework for supervised domain adaptation is created.
- *d-SNE*^56^: d-SNE is a novel technique method based on the distance metric that has achieved great transfer results on the image benchmark datasets.
- *DAS*$$^+$$^45^: This model employs two regularizations, entropy minimization and self-ensemble bootstrapping to refine its classifier while minimizing the domain divergence.
- *DAAN*$$^+$$^57^: DAAN is an adaptation network with dynamic adversarial.
- *SAFN*$$^+$$^58^: SAFN is the state-of-the-arts across many visual domain adaptation benchmarks.
- *ML*^42^: We apply the standard mutual learning in our task directly. ML can make the source domain and target domain collaborative and teach each other throughout the training process.

The original DANN, DAS, DAAN and SAFN are unsupervised domain adaptation models. As suggested in^59^, we use the source code of DANN, DAS, DAAN and SAFN and extend them to DANN$^+$, $\hbox {DAS}^+$, $\hbox {DAAN}^+$ and $\hbox {SAFN}^+$, which utilize target supervised information. They all have improved performances, respectively.

**Table 2:** Model comparison results. The state-of-the-art result of each evaluation indicator is bolded.

Systems		P	R	macro-F1	Acc
Non-Transfer	TextCNN	0.7543	0.7544	0.7543	0.7544
	TextRNN	0.7248	0.7237	0.7238	0.7243
	BiLSTM$+$Att	0.7625	0.7626	0.7625	0.7626
	TreeLSTM	0.7364	0.7279	0.7278	0.7317
	TreeGCN	0.8119	0.8045	0.8043	0.8057
	**CASA-T-D**	**0**.**8266**	**0**.**8282**	**0**.**8256**$^{\diamondsuit }$	**0**.**8259**$^{\diamondsuit }$

The marker $\diamondsuit$ refers to p-value $< 0.05$ when compared with TreeGCN in the paired t-test. All models run over five times with random initializations and are report average precision, recall, macro-F1, and accuracy.

### Implementation details

The word embeddings dimension is initialized with 200-dimensions. The GRU hidden layer size is 64 dimensions. The batch size is 64. The GCN hidden layers size is 128 dimensions, and the initial learning rate is 0.001. $\eta _{T}$ is not constant and $\eta _{D}$ and $\lambda$ are set in to be 0.1 and 0.9, respectively. Details on the hyperparameters are discussed in later sections. For a fair comparison of experimental results, feature extractors for all transfer learning models are set to CASA-T-D. Adam optimizer^60^ is used to train up to 30 epochs on the dataset, and the loss value is output every 100 batches. The training is stopped when the verification loss does not decrease for ten consecutive times.

### Main result analysis

#### Comparison with non-transfer

We compared the model in the context modeling stage with the current nontransfer model to explore the heterogeneous graph’s presentation ability in CASA. The results are shown in Table 2.

We note that (1) the CASA-T-D results are far better than those of the other models. Despite the same tree structure, the TreeGCN results are 7.4% higher than that of TreeLSTM, which shows that GCN captures depth features more effectively. (2) CASA-T-D and TreeGCN are both GCN convolutions but different in representation learning. In comparison, the result of CASA-T-D is 2.02% higher than that of TreeGCN. (3) The BiLSTM $+$ Att has a higher performance than TextRNN. One possible reason is that the attention mechanisms in text classification plays an important role. Thus, it could be more convincing that the CASA-T-D model has superior performance mainly due to the CAHG, which gathers rich semantic information.

**Table 3:** Model comparison results with domain adaptation.

Systems		Virus$\rightarrow$Target		Usual$\rightarrow$Target		Weibo$\rightarrow$Target		Hotel$\rightarrow$Target	
		F1	Acc	F1	Acc	F1	Acc	F1	Acc
Transfer	Fine-tuning	0.8192	0.8191	0.8093	0.8119	0.7966	0.7965	0.8272	0.8284
	$\hbox {DANN}^+$	0.8340	0.8345	0.8306	0.8314	0.8276	0.8284	0.8371	0.8386
	CCSA	0.8224	0.8232	0.8358	0.8376	0.8293	0.8304	0.8265	0.8273
	d-SNE	0.8189	0.8191	0.8349	0.8356	0.8300	0.8301	0.8202	0.8201
	$\hbox {DAS}^+$	0.8467	0.8469	0.8397	0.8396	0.8336	0.8335	0.7942	0.7955
	$\hbox {DAAN}^+$	0.8217	0.8099	0.8210	0.8109	0.8180	0.8078	0.8171	0.8068
	$\hbox {SAFN}^+$	0.8278	0.8284	**0**.**8461**	**0**.**8469**	0.8384	0.8397	0.8404	0.8407
	ML	0.8469	0.8479	0.8351	0.8366	0.8315	0.8325	0.8370	0.8376
	**CASA**	**0**.**8541**$^{\dagger }$	**0**.**8540**$^{\dagger }$	0.8433$^{\dagger }$	0.8448$^\dagger$	**0**.**8398**$^{\ddagger }$	**0**.**8407**$^{\ddagger }$	**0**.**8475**$^{\ddagger }$	**0**.**8489**$^{\ddagger }$

The state-of-the-art result of each dataset is bolded. The marker $\dagger$ refers to p-value $< 0.05$ by comparing with ML in paired t-test, while the marker $\ddagger$ refers to p-value $< 0.05$ by comparing with $\hbox {SAFN}^+$ in paired t-test. All models run over five times with random initializations and report the mean results.

**Table 4:** Ablation study results.

Systems		Accuracy
Best	CASA	**0**.**8541**
w/o CAHG	CASA*	0.8397
w/o Class adaptation	CASA-D	0.8376
w/o Domain adaptation	CASA-T	0.8335
w/o MGD	CASA-T-D	0.8259
w/o MGD&CAHG	CASA-T-D*	0.8181

For all transfer learning models, the source domain is virus. w/o CAHG means removing the sentence nodes and intergraph token-token links and turning them into a homogeneous graph structure for training. Accuracy is reported as the average result over 5 runs with random initialization.

#### Comparison with transfer

It can be observed from the experimental results in Table 3 that (1) for the popular technology “fine-tuning”, after adding Virus, Usual and Weibo source domain data, the target data accuracy rates dropped by 0.68%, 1.4%, and 2.94%, respectively. This resembles our predicted results because the feature distribution gaps in the source and target domains are too large. Fixed source domain parameters cannot be corrected sufficiently during fine-tuning; finally, negative migration occurs. (2) We try to apply advanced models in the visual DA to this task, but most of the results are not very outstanding. CCSA, d-SNE and $\hbox {DAAN}^+$ even showed negative transfer on individual datasets. For example, the accuracy of CCSA and d-SNE on Virus Target dropped by 0.27 and 0.68, respectively. This result may be caused by the diversity between the image and the natural language processing field. (3) The target domain experimental performances are improved with domain adaptation models CASA, $\hbox {DANN}^+$, $\hbox {DAS}^+$, $\hbox {SAFN}^+$ and ML for all source domains. This proves that the knowledge learned from explicit sentiment is helpful to the recognition of implicit sentiment.

(4) $\hbox {SAFN}^+$ has the outperformance result in the transfer of Usual $\rightarrow$ Target, which shows that many commonalities between visual domain adaptation and NLP domain adaptation could be mined. (5) In addition, the contribution of different source domains to implicit sentiment recognition is different. From Table 1, we know that the virus and hotel datasets are characterized by a small amount of data but a single topic of content. In contrast, the datasets Usual and Weibo have a large amount of data but on various topics. For CASA, the effect of single-topic transfer (Virus$\rightarrow$Target, Hotel$\rightarrow$Target) is better than that of multitopic transfer (Usual$\rightarrow$Target, Weibo$\rightarrow$Target). The Virus$\rightarrow$Target, with a more similar structure, has a better transfer effect than Hotel$\rightarrow$Target.

### Ablation study

To further compare each component CASA’s contribution, we sorted out the data of the ablation study part of the main experiment and plotted it into a line chart, as shown in Table 4. From Table 4, we can intuitively find the following information.

(1) Removing the CAHG, class discriminator, and domain discriminator of CASA separately, the experimental results drop by 1.44%, 1.65%, and 2.06% and are still higher than those of the nontransfer model CASA-T-D (82.59%). This shows that the fine-grained adjustment contributes to this transfer task, and the coarse-grained adjustment has greater performance than the heterogeneous structure. (2) When CASA-T-D removes CAHG and CASA* removes MGD, the accuracy drops by 0.78% and 2.16%, respectively, indicating that the CAHG and MGD we proposed can have a great impact on the experimental results.

**Figure 5:**
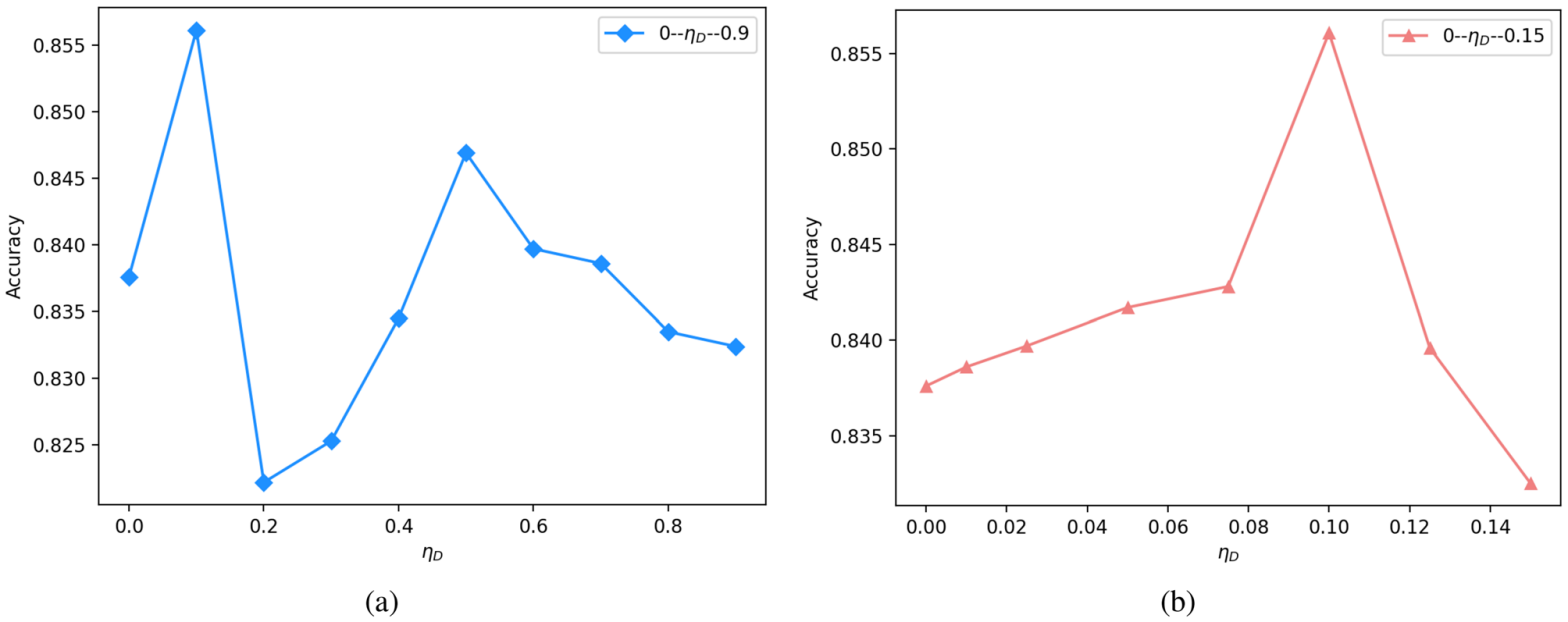
Hyperparameter study. For all $\eta _D$ values, the source domain is virus. Accuracy is reported as the average result over 3 runs with random initialization.

**Table 5:**
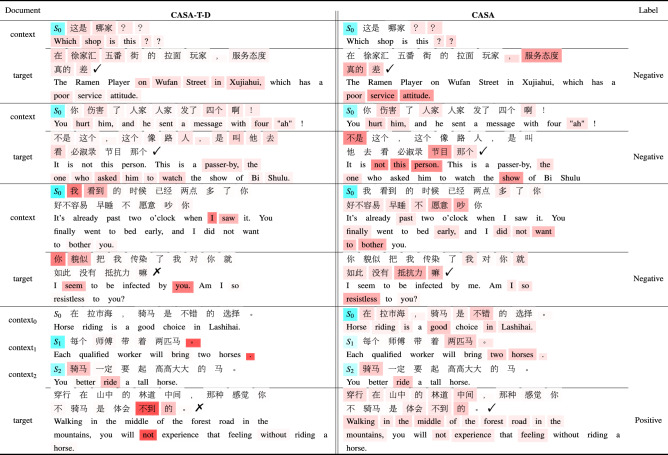
Case study. Visualization of attention weight distribution from CASA-T-D and CASA on testing examples. For CASA, the source domain is Virus. Marker $\checkmark$ signifies correct prediction, while marker × signifies incorrect prediction. Blue denotes the sentence weight, and red denotes the word weight.

### Hyperparameter study

In this section, we will present how to choose the value of hyperparameters $\eta _T$, $\eta _D$ and $\lambda$.

#### Hyperparameter $\eta _T$

Inspired by^5^, $\eta _T$ is not a constant, but changes from 0 to 1, namely $\eta _{T}=\frac{2}{1+\exp (-\alpha \cdot p)}-1$. Wherein, the hyperparameter $-\alpha$ in this paper is set to 10 as in^5^; The relative value *p* of the iterative process, that is, the current number of training steps / the total number of training steps, changes from 0 to 1 with the progress of training. The above formula means that at the beginning,$\eta _{T}= 0$ , the domain classification loss will not be passed back to the feature extractor network, and only the domain classifier is trained; As the training progresses, $\eta _{T}$ gradually increases, and the feature extractor is trained and begins to gradually generate features that can confuse the domain classifier.

#### Hyperparameters $\eta _D$ and $\lambda$

Which are selected through the validation set. First we have to judge whether $\eta _D$ and $\lambda$ is the same order of magnitude, and the order of magnitude after verification is $-1$.Therefore, set$\eta _D + \lambda = 1$ , When the verification set loss is minimum, checking the test accuracy.

Figure 5 shows the test set accuracy under different $\eta _D$ values. From (a), it shows that the optimal range of $\eta _D$ value is 0 to 0.2. Thus we did further experiments, as shown in (b). From (b), we can see that the optimal $\eta _D$ value is 0.10, so $\lambda$ is 0.90.

**Figure 6:**
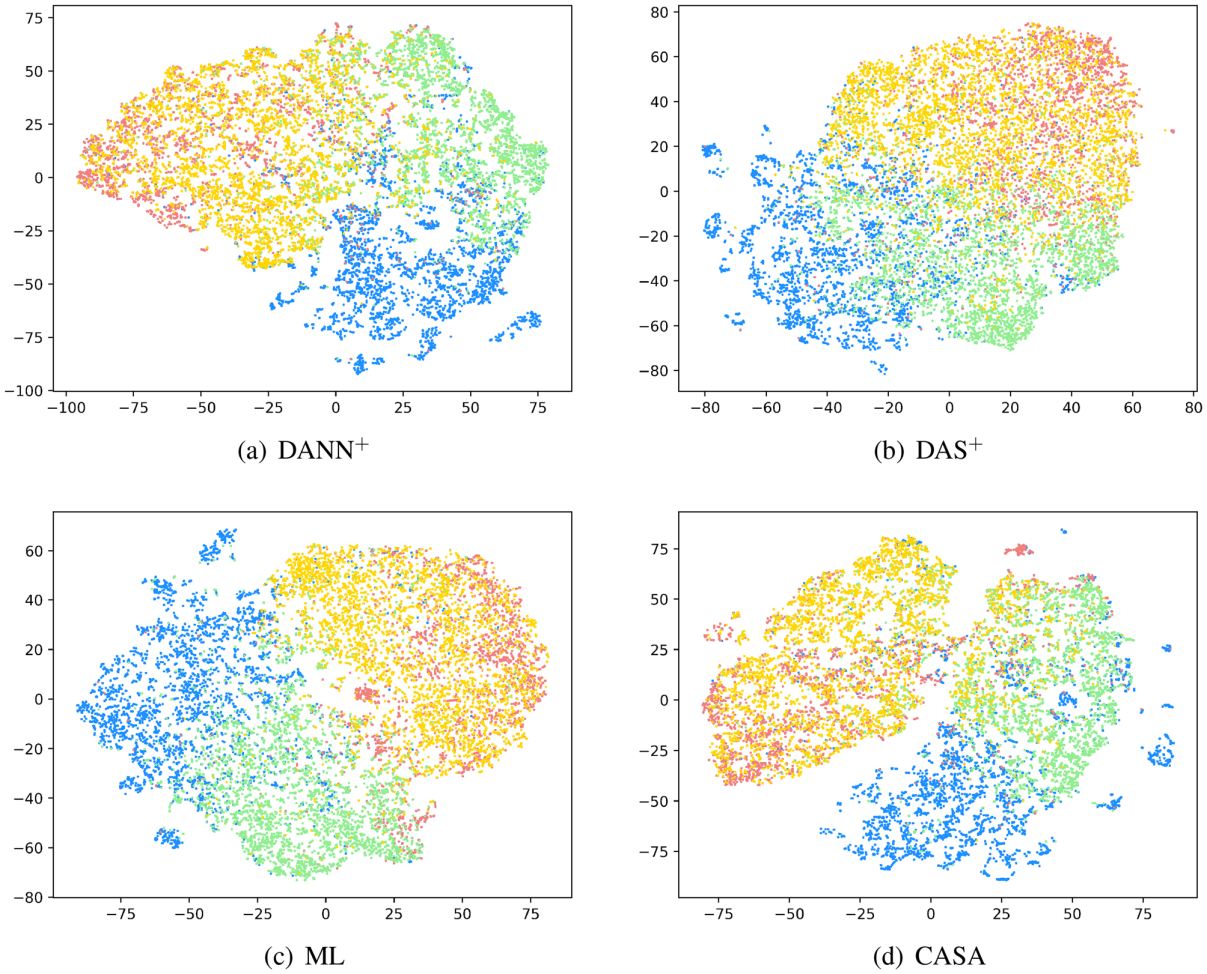
t-SNE visualization of the distribution of the features after different domain adaptation methods. Different colors indicate different sentiment polarities. (source domain: positive = dodger blue, negative = light coral; target domain: positive = light green, negative = gold).

## Effectiveness verification

### Case study

We want to explore what domain-specific information, which has been learned from the source domain, would enhance implicit emotion classification. In Table 5, we visualized the hierarchical attention layer in the nontransfer model CASA-T-D and the full model CASA. Using the CASA-T-D model as a benchmark, we compare the differences in attention weight distribution of different models under the same text.

The first two samples contain only one context and the target sentence. Although the two models’ attention scores are different, the critical points of emotional judgment are noted, such as ‘差 (poor)’ and ‘伤害 (harm)’. Obviously, these tokens are domain-independent words, which are usually used in many domains. In the third example, the CASA model learned the virus domain-specific information ‘抵抗力 (resistless)’, but CASA-T-D could not.

Then, we examine the impact of long contextual text on the two models. As shown in the last example, CASA-T-D focuses most of its attention on the token ‘不到 (not)’ in the target sentence and does not notice the token ‘不到 (good)’ in the context. In contrast, the CASA attention score is relatively scattered, conducive to the judgment of emotional polarity, which may benefit from source domain knowledge.

### Feature visualization

To better illustrate how the CASA works, we used t-SNE^61^ to reduce the dimensionality of the feature to two and visualize the data distributions after domain adaptation Virus $\rightarrow$ Target.

Figure 6 shows that the baseline models (a), (b) and (c) all virtually guarantee the source and target domain data fusion. On the other hand, the distance between the classes in the domain is still very close.

In contrast, benefitting from our proposed MGD, CASA’s intraclass boundary is significantly more significant, conducive to intraclass identification.

## Conclusions and future work

This paper proposes a CASA network via graph convolution for the cross-domain implicit sentiment classification problem, first building a relation between explicit and implicit sentiment. Existing studies either rarely consider using domain-specific semantic information or ignore maximizing class-specific decision boundaries. We aim to address the above two drawbacks. First, CASA provides a CAHG, effectively extracting domain-specific semantical information for both sources and targets. Hence, CASA improved the model’s generalization ability for implicit classification tasks. The case study shows that our model can effectively capture high-level domain-specific features. Second, CASA conducts an MGD to adapt the domain distribution, enhancing class distinction in each sentiment polarity decision boundary during domain adaptation. The feature visualization results show that CASA clarifies samples’ boundaries from different classes while adapting to the domain.

Moreover, there are several worthy challenges in cross-domain implicit sentiment tasks, such as transferring between the different single-domain topics, fine-grain sentiment transferring, ambivalence handling, and transferring explicit-to-implicit topics where the target domain tags are not given. We believe that all these factors can help us comprehend the link between explicit and implicit sentiment and that implicit sentiment analysis will be solved more effectively in the future.

## References

[1] Zhang, L. & Liu, B. Sentiment analysis and opinion mining. *Encycl. Mach. Learn. Data Min.* 1152–1161. 10.1007/978-1-4899-7687-1_907 (2017).

[2] Liu B (2012). Sentiment analysis and opinion mining. Synthesis Lect. Hum. Lang. Technol..

[3] Zuo E, Zhao H, Chen B, Chen Q (2020). Context-specific heterogeneous graph convolutional network for implicit sentiment analysis. IEEE Access.

[4] Zellinger, W., Lughofer, E., Saminger-Platz, S., Grubinger, T. & Natschläger, T. Central moment discrepancy (CMD) for domain-invariant representation learning. arXiv:arXiv:1702.08811 (2017).

[5] Ganin, Y. *et al.* Domain-adversarial training of neural networks. *Adv. Comput. Vis. Pattern Recognit.* 189–209. 10.1007/978-3-319-58347-1_10 (2017). arXiv:1505.07818.

[6] Li, Z., Zhang, Y., Wei, Y., Wu, Y. & Yang, Q. End-to-end adversarial memory network for cross-domain sentiment classification. *IJCAI Int. Joint Conf. Artif. Intell.* 2237–2243. 10.24963/ijcai.2017/311 (2017).

[7] Hu, M. *et al.* Domain-invariant feature distillation for cross-domain sentiment classification. *EMNLP-IJCNLP 2019 - 2019 Conference on Empirical Methods in Natural Language Processing and 9th International Joint Conference on Natural Language Processing, Proceedings of the Conference* 5559–5568, 10.18653/v1/d19-1558 (2020). arXiv:1908.09122.

[8] Peng, M., Zhang, Q., Jiang, Y. G. & Huang, X. Cross-domain sentiment classification with target domain specific information. in *ACL 2018—56th Annual Meeting of the Association for Computational Linguistics, Proceedings of the Conference (Long Papers)*. Vol. 1. 2505–2513. 10.18653/v1/p18-1233 (2018).

[9] Kipf, T. N. & Welling, M. Semi-supervised classification with graph convolutional networks. in *5th International Conference on Learning Representations, ICLR 2017—Conference Track Proceedings*. arXiv:1609.02907 (2019).

[10] Yao, L., Mao, C. & Luo, Y. Graph convolutional networks for text classification. *arXiv* (2018).

[11] Liu, X., You, X., Zhang, X., Wu, J. & Lv, P. Tensor graph convolutional networks for text classification. *arXiv* (2020).

[12] Zhang, C., Li, Q. & Song, D. Aspect-based sentiment classification with aspect-specific graph convolutional networks. *arXiv* (2019).

[13] Bai X, Liu P, Zhang Y (2021). Investigating typed syntactic dependencies for targeted sentiment classification using graph attention neural network. IEEE/ACM Trans. Audio Speech Lang. Process..

[14] Cambria E (2016). Affective computing and sentiment analysis. IEEE Intell. Syst..

[15] Chen Z (2015). Joint model for subsentence-level sentiment analysis with Markov logic. J. Assoc. Inf. Sci. Technol..

[16] Liu SM, Chen JH (2015). A multi-label classification based approach for sentiment classification. Expert Syst. Appl..

[17] Peng H, Ma Y, Poria S, Li Y, Cambria E (2021). Phonetic-enriched text representation for Chinese sentiment analysis with reinforcement learning. Inf. Fusion.

[18] Sun Y, Lin L, Yang N, Ji Z, Wang X (2014). Radical-enhanced Chinese character embedding. Lect. Notes Comput. Sci. (including subseries Lecture Notes in Artificial Intelligence and Lecture Notes in Bioinformatics).

[19] Chen, X., Xu, L., Liu, Z., Sun, M. & Luan, H. Joint learning of character and word embeddings. in*IJCAI International Joint Conference on Artificial Intelligence 2015—January*. 1236–1242 (2015).

[20] Yu, J., Jian, X., Xin, H. & Song, Y. Joint embeddings of Chinese words, characters, and fine-grained subcharacter components. in *EMNLP 2017—Conference on Empirical Methods in Natural Language Processing, Proceedings*. 286–291. 10.18653/v1/d17-1027 (2017).

[21] Peng, H., Cambria, E. & Zou, X. Radical-based hierarchical embeddings for Chinese sentiment analysis at sentence level. in *FLAIRS 2017—Proceedings of the 30th International Florida Artificial Intelligence Research Society Conference*. 347–352 (2017).

[22] Tao, H. *et al.* A radical-aware attention-based model for Chinese text classification. in *33rd AAAI Conference on Artificial Intelligence, AAAI 2019, 31st Innovative Applications of Artificial Intelligence Conference, IAAI 2019 and the 9th AAAI Symposium on Educational Advances in Artificial Intelligence, EAAI 2019*. 5125–5132. 10.1609/aaai.v33i01.33015125 (2019).

[23] Sorini C, Falcone M (2014). Jumping NLP curves: A review of natural language processing research. IEEE Comput. Intell. Mag..

[24] Yinxia, L. O., Zhang, Y., Fei, L. I., Qian, T. & Donghong, J. I. Emoji-based sentiment analysis using attention networks. *ACM Trans. Asian Low-Resour. Lang. Inf. Process.* **19**. 10.1145/3389035 (2020).

[25] Usama M (2020). Attention-based sentiment analysis using convolutional and recurrent neural network. Future Gener. Comput. Syst..

[26] Xi, C., Lu, G. & Yan, J. Multimodal sentiment analysis based on multi-head attention mechanism. *ACM Int. Conf. Proc. Ser.* 34–39. 10.1145/3380688.3380693 (2020).

[27] Yadav, R. K., Jiao, L., Goodwin, M. & Granmo, O. C. Positionless aspect based sentiment analysis using attention mechanism [formula presented]. *Knowl.-Based Syst.* **226**. 10.1016/j.knosys.2021.107136 (2021).

[28] Wang Z, Ho SB, Cambria E (2020). Multi-level fine-scaled sentiment sensing with ambivalence handling. Int. J. Uncertain. Fuzz. Knowl.-Based Syst..

[29] Hochreiter S, Urgen Schmidhuber J (1997). Long shortterm memory. Neural Comput..

[30] Dong, L. *et al.* Adaptive recursive neural network for target-dependent Twitter sentiment classification. in *52nd Annual Meeting of the Association for Computational Linguistics, ACL 2014—Proceedings of the Conference*. Vol. **2**. 49–54. 10.3115/v1/p14-2009 (2014).

[31] Kim, Y. Convolutional neural networks for sentence classification. in *EMNLP 2014—2014 Conference on Empirical Methods in Natural Language Processing, Proceedings of the Conference*. 1746–1751. 10.3115/v1/d14-1181. arXiv:1408.5882 (2014).

[32] Yang, Z. *et al.* Hierarchical attention networks for document classification. in *2016 Conference of the North American Chapter of the Association for Computational Linguistics: Human Language Technologies, NAACL HLT 2016—Proceedings of the Conference* 1480–1489. 10.18653/v1/n16-1174 (2016).

[33] Ma, D., Li, S., Zhang, X. & Wang, H. Interactive attention networks for aspect-level sentiment classification. in *IJCAI International Joint Conference on Artificial Intelligence*. 4068–4074. 10.24963/ijcai.2017/568 (2017).

[34] Wei J, Liao J, Yang Z, Wang S, Zhao Q (2020). BiLSTM with multi-polarity orthogonal attention for implicit sentiment analysis. Neurocomputing.

[35] Zhang X, Zhao J, Lecun Y (2015). Character-level convolutional networks for text classification. Adv. Neural Inf. Process. Syst..

[36] Conneau, A., Schwenk, H., Cun, Y. L. & Barrault, L. Very deep convolutional networks for text classification. in *15th Conference of the European Chapter of the Association for Computational Linguistics, EACL 2017—Proceedings of Conference*. Vol. 1. 1107–1116. 10.18653/v1/e17-1104 (2017).

[37] Paulus R, Socher R, Manning CD (2014). Global belief recursive neural networks. Adv. Neural Inf. Process. Syst..

[38] Teng Z, Zhang Y (2017). Head-lexicalized bidirectional tree LSTMs. Trans. Assoc. Comput. Linguist..

[39] Zhang, Y., Qi, P. & Manning, C. D. Graph convolution over pruned dependency trees improves relation extraction. in *Proceedings of the 2018 Conference on Empirical Methods in Natural Language Processing, EMNLP 2018*. 2205–2215. 10.18653/v1/d18-1244. arXiv:1809.10185 (2020).

[40] Mou, L., Li, G., Zhang, L., Wang, T. & Jin, Z. Convolutional neural networks over tree structures for programming language processing. in *30th AAAI Conference on Artificial Intelligence, AAAI 2016*. 1287–1293 . arXiv:1409.5718 (2016).

[41] Liao J, Wang S, Li D (2019). Identification of fact-implied implicit sentiment based on multi-level semantic fused representation. Knowl.-Based Syst..

[42] Zhang, Y., Xiang, T., Hospedales, T. M. & Lu, H. Deep mutual learning. in *Proceedings of the IEEE Computer Society Conference on Computer Vision and Pattern Recognition*. 4320–4328. 10.1109/CVPR.2018.00454. arXiv:1706.00384 (2018).

[43] Zhang, Y. *et al.* Every document owns its structure: Inductive text classification via graph neural networks. *arXiv*. 10.18653/v1/2020.acl-main.31. arXiv:2004.13826 (2020).

[44] Yosinski J, Clune J, Bengio Y, Lipson H (2014). How transferable are features in deep neural networks?. Adv. Neural Inf. Process. Syst..

[45] He, R., Lee, W. S., Ng, H. T. & Dahlmeier, D. Adaptive semi-supervised learning for cross-domain sentiment classification. in *Proceedings of the 2018 Conference on Empirical Methods in Natural Language Processing, EMNLP 2018*. 3467–3476. 10.18653/v1/d18-1383. arXiv:1809.00530 (2020).

[46] Qu, X., Zou, Z., Cheng, Y., Yang, Y. & Zhou, P. Adversarial category alignment network for cross-domain sentiment classification. in *NAACL HLT 2019—2019 Conference of the North American Chapter of the Association for Computational Linguistics: Human Language Technologies—Proceedings of the Conference*. Vol. 1. 2496–2508. 10.18653/v1/n19-1258 (2019).

[47] Bahdanau, D., Cho, K. H. & Bengio, Y. Neural machine translation by jointly learning to align and translate. in *3rd International Conference on Learning Representations, ICLR 2015—Conference Track Proceedings*. 1–15. arXiv:1409.0473 (2015).

[48] Ben-David S (2010). A theory of learning from different domains. Mach. Learn..

[49] Ben-David, S., Blitzer, J., Crammer, K. & Pereira, F. Analysis of representations for domain adaptation. *Adv. Neural Inf. Process. Syst.* 137–144. 10.7551/mitpress/7503.003.0022 (2007).

[50] Qian-Sun. A two-stage weighting framework for multi-source domain adaptation. *Adv. Neural Inf. Process. Syst.* 505–513 (2011).

[51] Chen, H. Y. & Chen, H. H. Implicit polarity and implicit aspect recognition in opinion mining. in*54th Annual Meeting of the Association for Computational Linguistics, ACL 2016—Short Papers*. 20–25. 10.18653/v1/p16-2004 (2016).

[52] Liu, P., Qiu, X. & Xuanjing, H. Recurrent neural network for text classification with multi-task learning. in *IJCAI International Joint Conference on Artificial Intelligence* 2016—January. 2873–2879. arXiv:1605.05101 (2016).

[53] Zhou, P. *et al.* Attention-based bidirectional long short-term memory networks for relation classification. in *54th Annual Meeting of the Association for Computational Linguistics, ACL 2016—Short Papers*. 207–212. 10.18653/v1/p16-2034 (2016).

[54] Tai, K. S., Socher, R. & Manning, C. D. Improved semantic representations from tree-structured long short-Term memory networks. in *ACL-IJCNLP 2015—53rd Annual Meeting of the Association for Computational Linguistics and the 7th International Joint Conference on Natural Language Processing of the Asian Federation of Natural Language Processing, Proceedings of the Conference*. Vol. 1. 1556–1566 (2015).

[55] Motiian, S., Piccirilli, M., Adjeroh, D. A. & Doretto, G. Unified deep supervised domain adaptation and generalization. *arXiv* (2017).

[56] Xu X, Zhou X, Venkatesan R, Swaminathan G, Majumder O (2019). D-SNE: Domain adaptation using stochastic neighborhood embedding. Proc. IEEE Comput. Soc. Conf. Comput. Vis. Pattern Recognit..

[57] Yu, C., Wang, J., Chen, Y. & Huang, M. Transfer learning with dynamic adversarial adaptation network. in *Proceedings—IEEE International Conference on Data Mining, ICDM* 2019—November. 778–786. 10.1109/ICDM.2019.00088. arXiv:1909.08184 (2019).

[58] Xu, R., Li, G., Yang, J. & Lin, L. Larger norm more transferable: an adaptive feature norm approach for unsupervised domain adaptation. in *Proceedings of the IEEE International Conference on Computer Vision* 2019—October. 1426–1435. 10.1109/ICCV.2019.00151. arXiv:1811.07456 (2019).

[59] Li, Z., Wei, Y., Zhang, Y., Zhang, X. & Li, X. Exploiting coarse-to-fine task transfer for aspect-level sentiment classification. in *Proceedings of the AAAI Conference on Artificial Intelligence*. Vol. **33**. 4253–4260. 10.1609/aaai.v33i01.33014253. arXiv:1811.10999 (2019).

[60] Kingma, D. P. & Ba, J. L. Adam: A method for stochastic optimization. in *3rd International Conference on Learning Representations, ICLR 2015—Conference Track Proceedings*. arXiv:1412.6980 (2015).

[61] Van Der Maaten L, Hinton G (2008). Visualizing data using t-SNE. J. Mach. Learn. Res..

